# Toxicity of Zero- and One-Dimensional Carbon Nanomaterials

**DOI:** 10.3390/nano9091214

**Published:** 2019-08-28

**Authors:** Iruthayapandi Selestin Raja, Su-Jin Song, Moon Sung Kang, Yu Bin Lee, Bongju Kim, Suck Won Hong, Seung Jo Jeong, Jae-Chang Lee, Dong-Wook Han

**Affiliations:** 1 Monocrystalline Bank Research Institute, Pusan National University, Busan 46241, Korea; 2 Department of Cogno-Mechatronics Engineering, College of Nanoscience & Nanotechnology, Pusan National University, Busan 46241, Korea; 3 Dental Life Science Research Institute & Clinical Translational Research Center for Dental Science, Seoul National University Dental Hospital, Seoul 03080, Korea; 4 GS Medical Co., Ltd., Cheongju-si, Chungcheongbuk-do 28161, Korea; 5 Bio-Based Chemistry Research Center, Korea Research Institute of Chemical Technology, Ulsan 44429, Korea

**Keywords:** carbon nanomaterials, unique properties, biomedical applications, in vitro toxicity, in vivo toxicity

## Abstract

The zero (0-D) and one-dimensional (1-D) carbon nanomaterials have gained attention among researchers because they exhibit a larger surface area to volume ratio, and a smaller size. Furthermore, carbon is ubiquitously present in all living organisms. However, toxicity is a major concern while utilizing carbon nanomaterials for biomedical applications such as drug delivery, biosensing, and tissue regeneration. In the present review, we have summarized some of the recent findings of cellular and animal level toxicity studies of 0-D (carbon quantum dot, graphene quantum dot, nanodiamond, and carbon black) and 1-D (single-walled and multi-walled carbon nanotubes) carbon nanomaterials. The in vitro toxicity of carbon nanomaterials was exemplified in normal and cancer cell lines including fibroblasts, osteoblasts, macrophages, epithelial and endothelial cells of different sources. Similarly, the in vivo studies were illustrated in several animal species such as rats, mice, zebrafish, planktons and, guinea pigs, at various concentrations, route of administrations and exposure of nanoparticles. In addition, we have described the unique properties and commercial usage, as well as the similarities and differences among the nanoparticles. The aim of the current review is not only to signify the importance of studying the toxicity of 0-D and 1-D carbon nanomaterials, but also to emphasize the perspectives, future challenges and possible directions in the field.

## 1. Introduction

**Figure 1:**
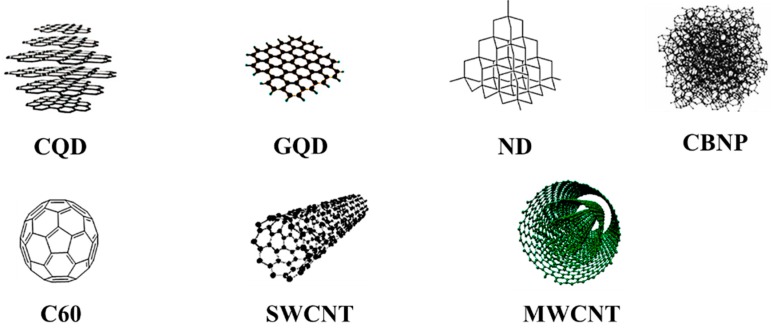
The structure of zero- and one-dimensional carbon nanomaterials have been shown. Carbon quantum dot (CQD) and graphene quantum dot (GQD), reproduced with permission from [11], Copyright Royal Society of Chemistry, 2010; nanodiamond (ND) and fullerene (C60), reproduced with permission from [7], Copyright American Chemical Society, 2013; carbon black nanoparticle (CBNP), reproduced with permission from [28], Copyright Elsevier, 2014; single-walled carbon nanotube (SWCNT) and multi-walled carbon nanotube (MWCNT), reproduced with permission from [29], Copyright Elsevier, 2017.

Nanotechnology has been a rapidly developing field, producing many nanomaterials with alterations in different physical and physicochemical properties such as size, shape, crystalline nature, and interaction with biological systems [1,2,3]. These materials have found adaptability in biomedical applications such as nanomedicines, cosmetics, bioelectronics, biosensors, and biochips [4]. However, the fact that possible health risks are associated with the increasing development of nanotechnology cannot be set aside. Nanoparticles may be either organic or inorganic based on the composition of elements. Mostly, inorganic nanomaterials are based on transition metals such as silver, iron, gold, zinc, copper, etc. whereas carbon nanomaterials are mainly composed of the carbon element, which constitutes various spatial arrangements in different nanoscales from zero (0-D) to three dimensions (3-D) [1,5,6,7]. In the present review, we will discuss the toxicity of 0-D carbon nanostructures (carbon black, nanodiamond, carbon nanodots and fullerene) and 1-D nanomaterials (single and multi-walled carbon nanotubes) from the research that has been conducted over the past two decades. The structure of carbon nanomaterials is shown in Figure 1.

Carbon dots are carbon-based nanomaterials with unique properties such as chemical inertness, optical stability, and wavelength-dependent photoluminescence [8]. Carbon quantum dots (CQDs) are typically quasi-spherical nanoparticles with a diameter less than 10 nm and composed of carbon, oxygen, hydrogen, nitrogen, and other elements. Because of their hydrophilic nature and cell permeation, CQDs have replaced traditional metal-based quantum dots in many applications, including photovoltaics, photocatalysis, and drug targeting [9]. The oxidized CQDs may contain 5–50% oxygen depending on synthetic procedures. Carbon quantum dots typically present two optical absorption bands in the UV-vis spectrum, which are attributed to π–π* and n–π* transitions in C=C and C=O bonds, respectively [10]. When the carbon nanodots are represented as a π-conjugated single sheet, with a size of 2–10 nm, they are called graphene quantum dots [11]. It has been reported that graphene quantum dots (GQDs) exhibit magnetic, electronic, and optical properties [12].

Nanodiamonds (NDs) are carbon-based crystalline nanoparticles inheriting diamond structure at the nanoscale with excellent properties such as optical transparency, hardness and chemical inertness [13]. The sp^3^ tetrahedral structure of the nanodiamond presents Raman signal at 1332 cm^−1^ and is capable of fluorescing due to point defects. However, the non-fluorescing nanodiamond displays a strong coherent anti-Stokes Raman scattering effect [14]. The quantitative analysis of cellular uptake of NDs is promising for the applications of bio labeling and bio imaging. The oxidized form of the nanodiamond has been reported to damage DNA in embryonic stem cells [15].

Carbon black nanoparticles (CBNPs) are the zero-dimensional carbon-based nanomaterials, which are produced in large quantities in different ways, such as partial combustion and thermal decomposition of hydrocarbons either in liquid or gaseous state [16]. The poor water-soluble carbon black poses a threat to health when exposed to the lungs through inhalation. The core portion of the insoluble particle yields reactive oxygen species (ROS), which render toxicity to the experimental animals [17]. Recently, the International Agency for Research on Cancer (IARC) listed carbon black nanoparticles as carcinogenic to human beings [16]. In toxicological studies, carbon black nanoparticles (CBNPs), with diameters less than 100 nm, have been reference material for diesel exhaust particles [18]. The aciniform aggregates of carbon black are basically fine powder in the size range of 100–1000 nm in a closed reaction chamber and form larger agglomerates due to van der Waals forces in the final step of the manufacturing process [19]. The term ‘carbon black’ should not be confused with such words as black carbon and soot, which are the carbonaceous materials emitted from incomplete combustion of fuels, such as waste oil, diesel, gasoline, wood, paper, plastic and rubber [20]. It is important to note that carbon black nanoparticles have certain physicochemical properties in common with another insoluble carbonaceous material, including graphene [16]. CBNPs have been widely used as conductive fillers due to their low aspect ratio, being economically inexpensive, and having good conductivity [21,22].

Among the carbon-based nanomaterials, fullerene (C60) is a generic term for a cluster composed of 60 carbon atoms that appears as a soccer-ball structure. The C60 contains 30 carbon atoms to readily interact with free radicals, and therefore is known as a free radical sponge [23,24]. The versatile applications of C60 include use in superconducting devices, energy device materials and catalysts [25]. The water-soluble polyhydroxylated fullerene, known as fullerenol (C60(OH)n), has been explored for its potential as being an anticancer, anti-HIV and skin rejuvenating cosmetic [25,26]. Fullerenol was reported to protect experimental animals from hepatotoxicity and doxorubicin-induced cardiotoxicity [26,27]. In nature, fullerene is available as its analogues including C70, C80, and C94, because of its tendency to aggregate and form a crystal-like structure with a diameter of 100 nm [23]. The research studies revealed that skin contact and nasal inhalation are the most likely routes of exposure to fullerenes for the workers in industries [25].

The unique property of CNT is its high aspect ratio, which promotes its superior properties to the encapsulating matrix polymers and has advantages over traditional reinforcements [30]. The most widely used techniques for the synthesis of carbon nanotubes (CNTs) are laser furnace, chemical vapor deposition, and arc discharge [31]. Their biomedical applications include biosensors, orthopedic prostheses, anticancer therapy, and tissue engineering [32]. The literature reports reveal that maternal exposure of CNTs might develop developmental toxicity such as teratogenicity [33]. The threat of nanotoxicity of CNTs is an increasing trend, as the global production of CNTs reaches several thousand tons per year [32]. Based on morphology, the carbon nanotube is generally classified into the two viz. single-walled and multi-walled carbon nanotubes. When one or several graphene sheets are rolled up to a cylindrical form concentrically, they yield single-walled carbon nanotubes (SWCNTs) and multi-walled carbon nanotubes (MWCNTs), respectively. Meanwhile, MWCNTs differ from SWCNTs in some physicochemical properties, such as the number of layers, the surface area and width [34,35]. The preparation of both CNTs also varies with different experimental conditions. For example, in the electric arc discharge method, SWCNTs are synthesized in the form of soot when a graphite rod comprising a metal catalyst acts as an anode and pure graphite as a cathode. Meanwhile, the production of MWCNTs is achieved strictly in the presence of inert gas such as helium. In the laser vaporization method, generation of SWCNTs mainly depends on the type of metal catalyst and the furnace temperature, whereas the yield of MWCNTs requires a pure graphite target and an optimum temperature of 1200 °C [36]. The nanotubes strongly interact with each other by van der Waals forces and hence exhibit hydrophobicity, which limits their biomedical applications. Hypochlorite, myeloperoxidase, and eosinophils peroxidase have been reported to degrade nanotubes within phagosomes and in the inflammation sites [37]. Researchers have adopted different approaches to modify pristine CNTs to impart hydrophilic behavior. The π-conjugated skeleton of CNT was covalently modified through different chemical reactions such as sidewall halogenation, hydrogenation, plasma activation, cycloaddition, radical, nucleophilic and electrophilic additions. The non-covalent modification occurs by physical attachment of various functional molecules and the endohedral filling takes place at the inner empty cavity of CNT [38].

SWCNTs have been used in a wide range of commercial applications such as earthquake-resistant buildings, dent-resistant car bodies, stain-resistant textiles and transistors [39]. The diameter of SWCNTs is approximately 1–2 nm and their toxicity is more substantial in comparison to MWCNTs (10–20 nm) and other carbonaceous nanomaterials such as carbon black and fullerene [40]. Despite being an attractive structural material with a high aspect ratio of length to width, carbon nanotubes threaten living organisms with potentially hazardous effects [41]. As far as the drug administration of SWCNTs is concerned, the inhalation route of exposure has more serious effects than the aspiration route in terms of oxidative stress, inflammatory responses, fibrosis and collagen deposition [42]. It has been reported that the agglomerates of SWCNTs caused granulomas in the proximal alveoli, and dispersed SWCNTs instigated interstitial fibrosis in the distal alveoli [43]. Similar to asbestos, MWCNTs have been reported to possess pathogenicity, owing to their larger durability and needle-like shape [32]. They found a wide variety of industrial applications in rechargeable batteries, water filters and sporting goods [44]. It was informed that non-branched MWCNTs had a higher potential to cause mesothelioma than the tangled MWCNTs [45].

## 2. In Vitro Cellular Toxicity of Zero- and One-Dimensional Carbon Nanomaterials

The in vitro toxicity effects of carbon nanomaterials (0-D and 1-D) have been listed in Table 1. The cytotoxic effect of the polyethylenimine (PEI) coated CQDs based nanohybrid, with a diameter of 6.5 ± 2 nm, was investigated at various concentrations (200, 400, 600 and 800 μg/mL) on kidney epithelial cells derived from the African green monkey. The MTT (3-(4,5-dimethylthiazol-2-yl)-2,5-diphenyltetrazolium bromide) assay revealed that the nanohybrid killed 39% of cells at concentration 600 μg/mL, despite there being no sign of significant toxicity at lower concentrations [46]. The pristine fluorescent carbon quantum dots (~7 nm) were evaluated for its cytotoxicity assessing total ROS, glutathione, and lactate dehydrogenase activity on human bronchial epithelial cells (16 HBE). The data revealed that CQDs preferentially located on the surface of cells and that its exposure induced oxidative stress and decreased cell viability [47]. A comprehensive study was presented to describe the critical role of functionalized nanoparticles in cytotoxicity using mouse embryonic fibroblasts (NIH-3T3). The CQDs synthesized from candle soot were negatively charged. The pristine CQDs were then functionalized with PEG (polyethylene glycol) and PEI to impart neutral and positive charges on the surface of nanoparticles, respectively. The results of in vitro cellular toxicity measurements revealed that the neutral charged CQDs did not induce any abnormalities in the cell cycle, cellular trafficking and cell morphology up to the concentrations of 300 µg/mL. Meanwhile, the negatively charged pristine CQDs arrested the cell cycle at the G2/M phase, enhanced cell proliferation, and caused oxidative stress. Being the most cytotoxic, the positively charged CQDs triggered a significant alteration in the cell cycle at the G0/G1 phase, at a concentration of 100 µg/mL [48].

GQDs have also shown different cellular uptake in MC3T3 osteoblast cell lines derived from mouse calvaria and exhibited low cytotoxicity due to their small size and high oxygen content [49]. The adverse effects of hydroxyl-modified GQDs (OH-GQDs) were studied on human lung carcinoma cell lines H1299 and A549. The OH-GQDs with hydrodynamic diameter of 10.3 ± 1.9 nm, at a concentration 50 μg/mL, decreased cell viability and intracellular ROS generation at a significant level. The cell signaling pathway analysis exposed that hydroxylated GQDs induced G0/G1 arrest, cell senescence, and inhibition of Rb phosphorylation in both types of cells [50]. It was confirmed that GQDs were less cytotoxic to human breast cancer (MCF-7) and human gastric cancer (MGC-803) cells on prolonged incubation. The nanoparticles significantly permeated into both cytoplasm and nucleus of the cells following caveolae-mediated endocytosis, but they did not affect cellular morphology. In addition, the nanoparticles exhibited lower cytotoxicity to MGC-803 cells when compared to MCF-7 cells [51].

Genotoxicity of NDs was analyzed on mouse embryonic stem cells and the results revealed that NDs of 4–5 nm expressed an elevated level of DNA repair proteins such as p53 and MOGG-1. Further, oxidized NDs were described to have more influence on triggering DNA damage than the pristine NDs. However, it was demonstrated that NDs, either in oxidized form or pristine, were not severe in toxicity when compared to MWCNTs [52]. Intracellular ROS, mitochondrial activity, apoptosis, colony formation, and cellular uptake were studied to provide elucidative information about the toxicity of NDs in two different cell lines HaCaT and A549. At concentration of 1.0 mg/mL, inhibition of colony formation and small degree apoptosis were observed in cells. However, it was found that NDs did not have any significant influence on cell viability and ROS production [53]. Treated with RAW 264.7 murine macrophages, the cytotoxicity of NDs were examined in various sizes (6–500 nm) and concentrations (0–200 μg/mL). Cell proliferation and metabolic activity were found reduced in a concentration dependent manner. Flow cytometry analysis revealed that the nanoparticles caused necrosis, leading to significant cytotoxicity, irrespective of particle size [54]. In vitro toxicity measurements were carried out in human blood cells and the reports exposed that NDs could change the kinetics of active oxygen production, cause erythrocyte hemolysis and destruct white cells [55].

The in vitro genotoxic and mutagenic potential of NDs were investigated in human lymphocytes and the nanoparticles were reported to inhibit cell proliferation-inducing apoptotic cell death above 50 μg/mL. The cellular oxidative stress generated by the nanoparticles was found to be dose-dependent. Significant changes in chromatin stability followed by DNA oxidative damage were established, even at a concentration of 1 μg/mL. NDs had the potential to stimulate micronuclei augmenting centromeric signals at 10 μg/mL [56]. The viability of human umbilical vein endothelial cells (HUVEC-ST) was investigated following the treatment of NDs, which was synthesized by the detonation method. The results of the MTT assay revealed that NDs showed a concentration-dependent cytotoxicity and ROS production in cells [57]. In a study, the cytotoxicity effect of nanodiamond particles was explored by correlating different surface functional groups on the nanoparticles, such as –OH, –COOH and –NH_2_. It was shown that NDs were cytotoxic to HEK293 cells when the concentration was above 50 μg/mL. The cationic nanodiamond had the potential to permeate negatively charged cell membrane and hence exhibited cytotoxicity. In addition, carboxylated nanodiamond (ND–COOH) was reported to possess embryotoxicity as well as teratogenicity [58].

The in vitro toxicity effect of CBNPs (260 ± 13.7 nm) was evaluated on A549 human alveolar basal epithelial cells and suggested that ultrafine particles induced a greater oxidative stress with prolonged inhibitory effects than fine particles [59]. Printex 90, a commercial name of carbon black nanoparticles with a diameter of 14 nm, exhibited an oxidative damage response in HepG2 cells at 25 mg/L, which was revealed from formamidopyrimidine DNA glycosylase (Fpg)-modified comet assay [60]. In another comet (Fpg) assay, it was discovered that an increased level of oxidized purines was observed when the nanoparticles were investigated in the FE1-MML Muta Mouse lung epithelial cell line. The mutant frequency was noticed in carbon black exposed cells following eight repeated 72 h incubations with a cumulative dose of 6 mg nanoparticles [61]. The western blot analysis exposed that ultrafine carbon black nanoparticles, at 30.7 μg/cm^2^, stimulated proliferation of human primary bronchial epithelial cells through oxidative stress and epidermal growth factor-mediated signaling pathway [62]. The cytotoxic and genotoxic effects of CBNPs were investigated on the mouse macrophage cell line RAW 264.7. The particle size and specific surface area was 14 nm and 300 m^2^/g, respectively. The data confirmed acentric chromosome fragments at all concentrations and there was a slight increase in micronuclei frequencies at 3 and 10 mg/L [63]. It was reported that CBNPs (100 μg/mL) could induce DNA single-strand breaks and induce AP-1 and NFκB DNA binding in A549 lung epithelial cell line after 3 h of exposure [64]. The toxicity measurements of CBNPs in THP-1 derived monocytes and macrophages exemplified that the nanoparticles supported endothelial activation and lipid accumulation in THP-1 derived macrophages. In addition, the nanoparticles influenced increased cytotoxicity, LDH levels and intracellular ROS production in a dose-dependent manner [65].

It was discovered that C60 fullerene of approximately 0.7 nm was less toxic than carbon black and diesel exhaust particles when FE1-MutaMouse lung epithelial cells were exposed to nanoparticles. The results of the comet assay revealed that C60 significantly increased the quantity of formamidopyrimidine-glycosylase sites (22%) and oxidized purines (5%), though the nanoparticles did not involve breaking DNA strands [66]. Genotoxic effects of C60 sized 0.7 nm were investigated by micronuclei test in the human lung cancer cell line (A549) at a concentration range of 0.02–200 μg/mL and increased micronuclei frequencies were observed in nanoparticles treated cells in a dose-dependent manner [67]. The genotoxic studies of colloidal C60 in human lymphocytes had shown genotoxicity at 2.2 μg/L, whereas the ethanolic solution of C60 had exhibited the same at 0.42 μg/L [68]. The polyhydroxylated C60 fullerenol presented a dose-dependent decrease in micronuclei frequency and chromosome aberration when the nanoparticles were treated with Chinese hamster ovary cells (CHO K1). However, the study did not show any genotoxic effects in the concentrations of 11–221 μm [27]. The cytotoxicity of hydroxylated fullerene was analyzed in vascular endothelial cells at different concentrations, 1–100 μg/mL, and a dose-dependent decrease in cell viability was perceived. Furthermore, it was reported that fullerenes affected cell growth and cell attachment with the potential to cause cardiovascular disease after a long period of exposure (10 days) [69].

The toxicity effect of SWCNTs was explored on human embryonic kidney cells (HEK293T) and reported that the nanoparticle exposure resulted in a decrease in cell adhesion, inhibition in cell proliferation and induction in apoptosis, depending on the dosage and time. In addition, a nodular structure was formed due to the nanoparticle aggregation and overlap of cells [70]. The agglomeration of CNTs had a larger impact on triggering cellular toxicity in human MSTO-211H cells. It was found that the agglomerated CNTs were more toxic compared to monodispersed CNTs [71]. The geometric structure of the nanoparticles played a pivotal role in determining cytotoxicity. A comparative study was provided in describing cytotoxicity of SWCNTs, MWCNTs, and C60 fullerenes on guinea pig alveolar macrophages. The order of displaying toxicity was as follows, SWCNTs > MWCNTs > C60 fullerenes [72]. The intracellular distribution of functionalized SWCNTs was studied in murine 3T3 and human 3T6 fibroblast cells. The length of the nanotube varied from 300 to 1000 nm and the outer diameter was 1 nm. The analyses revealed that SWCNTs resided either in the cytoplasm or nucleus after crossing the cell membrane, and exhibited toxicity when the concentration of nanoparticles reached above 10 µM [73]. It was confirmed that exposure of SWCNTs induced cutaneous and pulmonary toxicities in human bronchial epithelial cells (BEAS-2B) and human keratinocyte cells (HaCaT). The microarray analysis revealed that the nanoparticles triggered alteration of genes followed by transcriptional responses. Cellular morphology, integrity and ultrastructure were affected as the nanoparticles depleted antioxidants in the cells [74,75]. Functionalization of the nanoparticles had taken advantage of reducing the toxic level of nanoparticles. The derivatized SWCNTs were reported to have fewer toxic effects than pristine SWCNTs from in vitro cytotoxicity measurements in human dermal fibroblasts [76]. The introduction of SWCNTs into normal and malignant human mesothelial cells produced ROS causing cell death, DNA damage and H2AX phosphorylation [77]. It was reported that SWCNTs, with a primary particle size of 0.4–1.2 nm and specific surface area of 26 m^2^/g, had the potential to induce DNA damage in lung V79 fibroblasts [78].

The cytotoxic and genotoxic effects of single and multi-walled CNTs were studied on the mouse macrophage cell line RAW 264.7, and it was demonstrated that the exposure of nanoparticles stimulated ROS release, chromosomal aberrations, necrosis, and apoptosis, but they did not cause any inflammatory responses. In addition, MWCNTs were reported to penetrate the cell membrane and reside in the nuclear envelope [63]. Electron microscopic studies indicated that highly purified MWCNTs expressed higher cytotoxic effects by damaging the plasma membrane of mouse macrophages (J774.1). It was found that the cytotoxicity of MWCNTs was significantly larger than crocidolite, a fibrous form of sodium iron silicate [79]. The higher concentrated MWCNTs caused a decrease in cellular viability and an increase in inflammation on prolonged exposure to human epidermal keratinocytes (HEK) cells. The nanoparticles had the potential to penetrate the cell membrane and change the expression level of various proteins. The nanoparticles were reported to be abundantly present within cytoplasmic vacuoles of the cells after cell permeation [80]. The toxicity of MWCNTs of approximately 30 nm was evaluated in human skin fibroblasts (HSF42) and the results revealed that the nanoparticles disrupted intracellular signaling pathways, causing an increase in apoptosis and necrosis, and activated the genes associated with cellular cycle regulation, metabolism, cellular transport, and stress response [81]. Interestingly, oxidized MWCNTs were described to exhibit more toxicity than pristine MWCNTs. Both were reported to induce apoptosis in T lymphocytes depending on the time period and dose [82].

## 3. In Vivo Toxicity of Zero-and One-Dimensional Carbon Nanomaterials

In some studies, the researchers performed in vivo animal studies of carbon nanomaterials after the careful evaluation of their in vitro toxicity measurements, and some of studies are listed in Table 2.

The toxicity of carbon quantum dots was investigated in different species such as zebrafish, zooplankton, and phytoplankton. The primary particle size was less than 10 nm, with interlayer spacing of 0.32 nm. It was found that zooplankton was more sensitive to CQDs than zebrafish and phytoplankton species and suffered oxidative stress, water acidification, insufficiency of nutrients and no photosynthesis in a time and dose-dependent manner [83]. When the nanoparticles were administered intravenously to ICR male and female mice with a single dose, it was observed that male mice are more sensitive than female mice, and that the nanoparticles treated male mice suffered severe acute inflammatory responses [84]. The intraperitoneal injection of CQDs (8 ± 2 nm) into male ICR mice affected cell membrane, immune system and liver clearance rate [8]. While investigating the in vivo toxicity of CQDs (2–6 nm) in embryos/larvae of male and female rare minnow, concentration-dependent embryos yolk agglutination, decreased spontaneous movements, and increased heart rate were observed [9]. The toxicity studies of GQDs in AB strains of wild-type zebrafish embryos/larvae revealed that the nanoparticle had the potential to decrease heart rate, causing disrupted embryonic development in a concentration dependent manner. However, the treatment of nanoparticles did not have significant toxicity at lower doses [85,86]. The toxicity of functional GQDs in an animal model was studied to understand the influence of functional groups attached on the surface of nanoparticles. The polyethylene glycol modified GQDs (PEG-GQDs) exhibited no significant toxicity when the nanoparticles were instilled intraperitoneally into female BALB/c mice [87]. Likewise, carboxylated GQDs (COOH-GQDs) triggered no obvious damage to SD rats after 21 days of intravenous post-administration [88].

The microinjection of NDs (0.5 mg/mL) to wild type young Caenorhabditis elegans had shown no detectable toxicity in brood size and longevity of animals. The hydrodynamic diameter of the nanoparticles in solution was approximately 120 nm [89]. When NDs of approximately 4 nm were intratracheally injected into male ICR mice at a concentration of 1.0 mg/kg, the nanoparticles produced lung burden during the whole exposure time, but there was no event of lipid peroxidation in lung tissue [90]. A dose-dependent toxicity was observed in the lung tissue of male Kun Ming mice after the NDs were intratracheally administered at different concentrations 0.8, 4.0 and 20 mg/kg [13]. While investigating possible toxicity of bovine serum albumin functionalized nanodiamond (ND-BSA, ~100 nm) in AB strain zebrafish embryos at a concentration range of 1–5 mg/mL and 4–96 h post-fertilization (hpf), it was found that the control and NDs treated groups had no significant differences in embryonic development at concentration of 1 mg/mL. However, a higher concentration of NDs affected the pharyngula stage of embryos and caused fin curve in larvae during the hatching stage [14].

There were many reports that demonstrated the toxicity of carbon nanomaterials in animal models, which included pulmonary inflammation, DNA breaks, oxidative stress and elevated expression of mRNAs [17,91,92,93,94,95,96,97,98,99,100,101,102,103,104,105,106]. The intratracheally administered CBNPs (67 µg/animal) to female pregnant mice did not trigger significant germline mutation when compared to the control [107]. When the rats were exposed to 7.1 and 52.8 mg/m^3^ of CBNPs for 13 weeks, a significant dose-dependent increase in hypoxanthine-guanine phosphoribosyltransferase (hprt) mutation frequency was observed in rat alveolar epithelial cells. The nanoparticles impaired lung clearance, causing lung burden, and changed the expression of bronchoalveolar lavage fluid (BALF) markers of inflammation and lung injury [108]. Various immunohistochemical measurements were established to quantify DNA damage markers such as poly (ADP-ribose), 8-hydroxyguanosine, and 8-oxoguanine DNA glycosylase after intratracheally instilling CBNPs into rats for 3 months. The analyses revealed that the nanoparticles had significantly increased the expression of DNA damage markers, though the genotoxicity was less pronounced [109]. Genotoxic effects, acute phase and inflammatory responses were examined while exposing C57BL/6JBomTac mice to CBNPs. Even at low exposure doses of nanoparticles (0.67, 2, 6 µg), an increase in DNA strand breaks occurred in bronchoalveolar lavage (BAL) cells. It was reported that DNA damage was triggered by primary genotoxicity without inflammatory responses [110]. The pulmonary toxicity of carbon black nanoparticles was studied in C57BL/6 female mice administering a single dose of 0.162 mg. An increase in expression of miRNAs such as miR-135b, miR-21, and miR-146b, which are associated with pulmonary inflammation, was observed [111]. The polycyclic aromatic hydrocarbon modified CBNPs (PAH-CBNPs) were demonstrated to express the noticeable amount of keratinocyte chemoattractant and IL-6 mRNA, when compared to uncoated CBNPs and air control when male Wistar rats were subjected to nasal inhalation exposure for 2 weeks at a concentration of 6 mg/m^3^. The primary particle size and specific surface area of functionalized CBNPs was 14.2 ± 0.1 and 115 ± 3 m^2^/g, respectively [94].

The toxicity of fullerene of 96 nm was studied after subjecting male Wistar rats to whole-body inhalation for 4 weeks. The experiment was carried out for 6h/day with the exposure of 0.12 mg/m^3^. No significant changes were reported in the gene expression of CINC-1, CINC-2αβ, and CINC-3 in lung tissue [95]. In another similar study, the upregulation of genes associated with inflammation, oxidative stress and apoptosis was noted after one month of nanoparticle exposure. The geometric mean diameter of fullerene nanoparticles was 96 nm and specific surface area of them was 0.92 m^2^/g [96]. The intratracheal instillation of C60 to gpt delta transgenic mice at a single dose of 0.2 mg/mouse induced mutant frequencies with 2–3-fold increase in comparison to the control. When administered at multiple doses (4 times), the nanoparticles brought about transversion of A:T to T:A in treated animals [67]. The intratracheally instilled C60 (46.7 ± 18.6 nm) increased the expression of pro-inflammatory cytokines including tumor necrosis factor-α (TNF-α), interleukins (IL-1 and IL-6) and T-cell distribution in ICR male mice [97]. It was demonstrated that single oral intragastric administration of fullerene to female Fisher 344 rats generated oxidative damage along with the expression of mRNA 8-oxoguanine DNA glycosylase (8-oxodG) in the lung at high dose [98]. No acute oral toxicity was reported for the C60 treated Sprague-Dawley male and female rats for 2 weeks [26]. The intratracheally administered fullerenol (C60(OH)_n_) showed increased neutrophil influx in the lungs causing inflammation in BALB/c female mice after 24 h of post-administration of 200 μg/mouse [99].

The DNA damage was examined in rats following intragastric instillation of SWCNT at a concentration of 0.64 mg/kg body weight. SWCNTs were demonstrated to elevate the levels of 8-oxodG in liver and lung tissues of rats. The length and width of the nanoparticles was less than 1 µm and 0.9–1.7 nm, respectively [98]. The aortic mitochondrial alteration was studied using oxidative stress assays in SWCNTs exposed C57BL/6 mice. The intra-pharyngeal instilled SWCNTs (40 μg/mouse) activated heme oxygenase 1, which is indicative of oxidative stress. The nanoparticles exhibited increased mitochondrial DNA damage accompanied by the changes in aortic mitochondrial protein carbonyl and glutathione levels [112].

The general toxicity effects of MWCNTs were inflammation, granuloma and fibrosis when in vivo toxicity measurements were performed in experimental animals [103,104,106]. The induction of mesothelioma in p53+/− mouse was studied by the intraperitoneal application of multi-wall carbon nanotube. It was found that intraperitoneally administered, micro-sized MWCNTs (10–20 µm) stimulated mesothelioma such as the positive control, crocidolite [113]. The immune and inflammatory responses of MWCNTs were tested following intraperitoneal administration of a single dose of 2 mg/kg body weight to female ICR mice. After 1 week of post-exposure, the expression of leukocyte adhesion molecules and cluster of differentiation on granulocytes were found increased. The number of monocytes, leukocytes, and granulocytes were also present in peripheral blood significantly. MWCNTs were reported to exhibit sustained immune responses with the overexpressed ovalbumin specific IgG1 and IgM. The original morphology of the liver had also suffered changes to a rounded shape along with the appearance of MWCNTs on internal organs [114].

## 4. Conclusions and Perspectives

In this review, we have discussed the toxicity effects of 0-D and 1-D carbon nanomaterials in different cell lines and animal models. It was demonstrated that differential toxicity of carbon nanomaterials was inherited from various factors such as size, dispersion, cell permeability, and functionalization. Though the researchers studied the toxicity of carbon nanomaterials in both in vitro and in vivo intensively, there are still some issues to be addressed. (1) Many researchers showed experimental results with the aim of comparing the toxicity of two or more carbon-based nanoparticles for the same cell line and animal model. A comparative study is required for different cell line sources and animal species for the same kind of nanoparticle. (2) There are many studies that emphasize the role of the encapsulating agents on the nanoparticles in altering the overall functionality. The differential toxicity depending on the charge on the surface of nanoparticles has also been demonstrated. However, a systematic study is needed to corroborate the toxicity results with the surface charge of the nanoparticles (either positive or negative) with subtle differences. (3) The toxicity studies of the same kind of carbon nanoparticle prepared from different techniques should also be examined. The following suggestions are put forth for future research in this field: (1) A comprehensive study on the toxicity of carbon nanomaterials using different physicochemical and biological parameters to exemplify toxicity limitation and prove the effectiveness of the materials. (2) A systematic study to ensure that the carbon nanoparticles exhibit toxicity towards cancerous cells but not normal cells at the established concentration range. Undoubtedly, the knowledge of the toxicity of carbon nanomaterials will help the researchers with interdisciplinary backgrounds to deliver more successful biocompatible materials to society in the future.

## Figures and Tables

**Table 1:** The in Vitro Toxicity Effects of 0-D and 1-D Carbon Nanomaterials.

Carbon Nanomaterial; Nanoparticle Dimension	Cell Line; Concentrations; Exposure	Toxicity Effects	Reference
PEI-CQDs; PS = 6.5 ± 2 nm, HD = 56.54 nm	Kidney epithelial cells (African green monkey); 200, 400, 600 and 800 μg/mL; 48 h	PEI-CQDs exhibited toxic effects above concentration 600 μg/mL.	[46]
CQDs; PS = ~7 nm, HD = 60.3 ± 7 nm	Human bronchial epithelial cells (16HBE); 1, 10, 50, 100 and 200 μg/mL; 24 h	CQDs reduced cell viability inducing oxidative stress.	[47]
OH-GQDs; PS = 5.6 ± 1.1 nm, HD = 10.3 ± 1.9 nm	Human lung carcinoma cell lines (H1299 and A549); 12.5, 25, 50 and 100 μg/mL; 24 and 48 h	The hydroxylated GQDs induced cell senescence and inhibited Rb phosphorylation in both types of cells at concentration 50 μg/mL.	[50]
GQDs; PS = ~20 nm	Human breast cancer cells (MCF-7) and human gastric cancer cells (MGC-803); 20, 100, 200 and 400 μg/mL; 24 h	GQDs were found less cytotoxic on both type of cells though the nanoparticles permeated into cytoplasm and nucleus.	[51]
NDs; PS = 4 –5 nm	Mouse embryonic stem cells; 5 or 100 μg/mL; 24 h	NDs exhibited genotoxicity, expressing an increased level of DNA repair proteins.	[52]
NDs; HD = 41–103 nm	Human keratinocyte (HaCaT) and human alveolar basal epithelial cells (A549); 0.01, 0.1 and 1.0 mg/mL; 6 and 24 h	NDs were not involved in decreasing cell viability and generating intracellular ROS. However, the nanoparticles inhibited colony formation in cells even at concentration 1.0 mg/mL.	[53]
NDs; PS = 6–500 nm	Mouse macrophages (RAW 264.7); 0, 10, 50, 100 and 200 μg/mL; 24 h	The results revealed that NDs reduced cell proliferation and metabolic activity in a dose dependent manner.	[54]
CBNPs; PS = 260 ± 13.7 nm	A549 cells; 0.39 and 0.78 μg/mL; 24 and 48 h	Size dependent cytotoxicity was observed in CBNPs treated cells. Ultrafine CBNPs affected more oxidative stress in cells than fine CBNPs.	[59]
CBNPs; PS = 14 nm	FE1-Muta mouse lung epithelial cell line; 75 μg/mL; 8 × 72 h	CBNPs caused genetic mutation increasing the quantity of oxidized purines.	[61]
CBNPs; PS = 14 nm, SSA = 300 m^2^/g	RAW 264.7 cells; 0.25, 10, 25, 50 and 100 μg/mL; 24, 48 and 72 h	Cytotoxic and genotoxic effects were observed, along with the formation of acentric chromosome fragments at all concentrations.	[63]
CBNPs; PS = 14 nm	A549 cells; 100 μg/mL; 0.5–24 h	CBNPs induced DNA single-strand breaks at 100 μg/mL at 3 h of post exposure.	[64]
C60; PS = 0.7 nm	FE1-Muta mouse lung epithelial cells; 100 μg/mL; 576 h	C60 increased the level of oxidized purines significantly without affecting DNA strands.	[66]
C60; PS = 0.7 nm	A549 cells; 0.02–200 μg/mL; 48 h	C60 treated cells witnessed increased micronuclei frequency depending on dosage.	[67]
C60(OH)n	Chinese hamster ovary cells (CHO K1); 11–221 μM; 24 h	The nanoparticles treated cells showed decreased micronuclei frequency and chromosome aberration in a dose dependent manner.	[27]
C60(OH)n; PS = 7.1 ± 2.4 nm	Human umbilical vascular endothelial cells; 1–100 μg/mL; 24 h	The hydroxylated C60 decreased cell viability in a concentration dependent manner.	[69]
SWCNTs; n/a	Human embryonic kidney cells (HEK293T); 0.78, 1.56, 3.12, 6.25, 12.5, 25, 50, 100, 150 and 200 μg/mL; 0–5 days	SWCNTs decreased cell adhesion and inhibited cell proliferation depending on dose and time.	[70]
SWCNTs; L = 300–1000 nm, W = 1 nm	Murine 3T3 and human 3T6 fibroblast cells; 1, 5 and 10 μM; 1 h	The nanoparticles had the potential to permeate the cell and exhibited toxicity above 10 µM.	[73]
SWCNTs; PS = 0.8–2.0 nm	Normal and malignant human mesothelial cells; 12.5, 25 and 125 μg/cm^2^; 24 h	DNA damage, cell death, and ROS generation were observed in nanoparticles treated cells.	[77]
SWCNTs; PS = 0.4–1.2 nm, SSA = 1040 m^2^/g	Chinese hamster lung V79 fibroblasts; 0, 24, 48 and 96 μg/cm^2^; 3 and 24 h	SWCNTs caused DNA damage in cells at 24 h of post-exposure.	[78]
MWCNTs; PS = 67 nm, SSA = 26 m^2^/g	Mouse macrophages (J774.1 and CHO-K1); 10–1000 μg/mL; 16–32 h	MWCNTs treated cells exhibited larger cytotoxicity than crocidolite treated cells.	[79]
MWCNTs; PS = 100 nm	Human epidermal keratinocytes (HEK) cells; 0.1, 0.2 and 0.4 mg/mL; 1, 2, 4, 8, 12, 24 and 48 h	MWCNTs penetrated the cell membrane and altered the gene expression level of various proteins.	[80]
MWCNTs; PS = 30 nm	Human skin fibroblasts (HSF42); 0.06, 0.6 and 6 μg/mL; 48 h	MWCNTs caused an increase in apoptosis and necrosis disrupting intracellular signaling pathways, cell metabolism and cellular transport.	[81]
MWCNTs; L = 1–5 µm, W = 20–40 nm	Human blood T lymphocytes; 10 ng/cell; 0, 24, 48, 72, 96 and 120 h	The oxidized form of MWCNTs exhibited more cytotoxicity than pristine MWCNTs. Both types of nanoparticles induced apoptosis in cells in a time and dose dependent manner.	[82]

Abbreviations: PS, particle size; HD, hydrodynamic diameter; SSA, specific surface area; L, length; W, width; n/a, not available.

**Table 2:** The in Vivo Toxicity Effects of 0-D and 1-D Carbon Nanomaterials.

Carbon Nanomaterial; Nanoparticle Dimension	Animal Model; Concentrations; Exposure	Toxicity Effects	Reference
CQDs; PS ≤ 10 nm, HD = 40 nm, IS = 0.32 nm	Zebrafish; 0, 10, 30, 50, 70, 100 and 200 mg/L; 0, 24, 48, 72, and 96 h Zooplankton; 0, 10, 30, 50, 70, 100 and 200 mg/L; 48 h Phytoplankton; 0, 5, 10, 50, 100, 200 and 500 mg/L; 0, 24, 48, 72, and 96 h	CQDs, at higher dose of 200 mg/L, did not affect swimming and feeding behaviors. CQDs exhibited moderate toxicity to zooplankton, inducing mortality and immobility with EC50 value 97.5 mg/L. CQDs induced oxidative stress and water acidification, inhibited photosynthesis and depleted nutrition absorption in a dose and time dependent manner. It retarded the growth of phytoplankton with EC50 value 74.8 mg/L at 96 h of the study.	[83]
CQDs; PS = 1–5 nm	Male and female ICR mice; 250, 320, 400 and 500 mg/kg, single dose, intravenous injections; 14 day sMale ICR mice; 100 mg/kg, repeated dose, intravenous injections; 1, 7, 30 and 90 days, once/day	Male mice (LD50 391.62 mg/kg) were found to be more sensitive to the higher doses of the nanoparticles than female mice (LD50 357.77 mg/kg). An acute inflammatory response was observed after seven doses, however the data on the body weight, organ coefficients, blood biochemistry, and organ histopathology suggested that the nanoparticles had low toxicity during the entire experimental period.	[84]
CQDs; PS = 2–6 nm	Male and female embryos/larvae of rare minnows; 0, 1, 5, 10, 20, 40, and 80 mg/L; 12–96 hpf	In lower dose treated groups (1, 5, 10, and 20 mg/L), no significant developmental defects were observed at the stage of 12 hpf, whereas higher dose treated groups (40 mg/L and 80 mg/L) caused embryos yolk agglutination in a concentration-dependent manner. The noticeable time-dependent deleterious effects were decreased spontaneous movements, higher heart rate, and increased hatching rate. Most of the unhatched embryos died when the exposure time reached 96 hpf.	[9]
CQDs; PS = 8 ± 2 nm	Male ICR mice; 0, 6, 12 and 24 mg/kg, intraperitoneal injection; 30 days	The histopathological examination showed that no obvious toxic effects were triggered by CQDs on mice. However, NMR metabolomic profiles revealed that CQDs could affect cell membrane, immune system, and normal liver clearance.	[8]
GQDs; PS = 2.3–6.4 nm, IS = 0.36 nm, height = 0.6–3.5 nm, 1–3 layers	AB strains of wild-type zebrafish embryo/larva; 0, 12.5, 25, 50, 100 and 200 μg/mL; 4–120 hpf	The heart rate of treated animals was found to be decreased with a dose-dependent effect. The exposure of GQDs suggested that they might have little effect during the heart development stage of zebrafish embryos and larvae.	[85]
GQDs, PS = 3.315 ± 1.74 nm	AB strains of wild-type zebrafish embryo/larva; 0, 12.5, 25, 50, 100, and 200 μg/mL; 4–96 hpf	At low concentrations of GQDs, no significant toxicity was observed. When the concentration was above 50 μg/mL, GQDs disturbed the embryonic development. The hatching rate and heart rate were decrease, accompanied with an increase in mortality. At high concentration of GQDs (200 μg/mL), various embryonic malformations including pericardial edema, vitelline cyst, bent tail, and bent spine occurred.	[86]
PEG-GQDs; PS = 3–5 nm, height = 0.5–1 nm, 1–2 layers	Female BALB/c mice; 20 mg/kg, intraperitoneal injection, multiple doses; 2 weeks	PEG-GQDs exhibited no-toxicity effects because of nanoparticle encapsulation.	[87]
COOH-GQDs; PS = 3–6 nm	SD rats; 5 and 10 mg/kg, intravenous injection; 7 doses in 22 days with an interval of 2 days	The studies revealed that the GQDs were distributed in liver, spleen, lung, kidney, and tumor sites after injection, however there was no obvious organ damage at 21 days of post-administration. The serum biochemistry and complete blood count studies revealed that the GQDs did not cause any significant toxicity to the treated animals.	[88]
NDs; HD = ~120 nm	Wild type young Caenorhabditis elegans; 0.5 mg/mL, microinjection	The NDs were found in the distal gonad and oocytes at 30 min after injection. No detectable toxicity effects were found in brood size and longevity of the treated animal groups.	[89]
NDs; PS = 4 and 50 nm, IS = 0.202 nm	Male ICR mice; 1.0 mg/kg, intratracheal instillation; 1, 7, 14 and 28 days of post-exposure	At 1 day of post-exposure, both kinds of nanoparticles produced a temporary increase in lung index but there was no trace of lipid peroxidation in lung tissue. During the whole exposure period, the burden of nanoparticle in macrophages was observed and the number of nanoparticles decreased by time in alveolar.	[90]
NDs; PS = 2–10 nm and 40–100 nm	Male Kun Ming mice; intratracheal instillation; 0.8, 4 and 20 mg/kg; 3 days	A dose-dependent toxicity effect was observed in the lung tissue of mice at 3 days of post-exposure of both kinds of nanoparticles and the higher concentration treated mice (4 and 20 mg/kg) exhibited significant toxicity.	[13]
NDs-BSA; PS = ~100 nm	Zebrafish (AB strain) embryos/larvae; 1, 2, 5 mg/mL; 4–96 hpf	The different stages of zebrafish embryos exhibited similar development when compared to the control groups at a lower concentration of NDs (1 mg/mL). However, a higher concentration of NDs affected the zebrafish embryos at the Pharyngula stage. The medium concentrated NDs (2 and 5 mg/mL) caused fin curving of zebrafish larvae at the hatching stage.	[14]
CBNPs; PS = 14 nm	Female C57BL/6J mice, 10 mg/mouse, intratracheal instillation; 21 days	CBNPs did not exert any significant adverse clinical effects. However, the histopathological studies revealed that they decreased lung compliance inducing inflammation when administered along with bleomycin. They augmented the levels of CCL2, TGF-b1, KC, IL-6, and nitrotyrosine in mice on different days of exposure.	[91]
CBNPs; PS = 14 and 56 nm	Male ICR mice; 50 μg/body, intratracheal instillation; 1, 7 or 14 days	CBNPs of 14 nm aggravated porcine pancreatic elastase mediated pulmonary exposure on emphysematous lung injury at an early stage (day 1) and expressed more interleukin-b and keratinocyte-derived chemoattractant. CBNPs of 56 nm caused inflammation but did not induce porcine pancreatic elastase triggered pathophysiology in the lung.	[92]
CBNPs; PS = 14 nm, SSA = 295–338 m^2^/g	Time mated C57BL/6BomTac mice, 42 mg/m^3^, whole-body inhalation; 1 h/day on gestation days (GD) 8–18 days 11, 54 and 268 μg/animal, intratracheal instillation; 1 h/day, GD 7, 10, 15 and 18 days	The whole-body inhalation induced significant DNA strand breaks in the liver of mothers and their offspring, whereas the intratracheal instillation did not have that effect. However, gestation and lactation were not affected in both ways of administrations. The pulmonary inflammation in time mated mice was similar in both administrations for the medium dose of nanoparticles.	[17]
CBNPs; PS = 14 nm, SSA = 295–338 m^2^/g	Female C57BL/6 mice; 162 μg/mouse, intratracheal instillation; 3 h, 1, 2, 3, 4, 5, 14 and 42 days	In the initial days of post-exposure, the worsening of pulmonary homeostasis occurred by the induction of oxidative stress, DNA strand breaks, cell cycle arrest, and cell death. Multiple chronic pulmonary inflammatory processes were the possible effects at the later points of post-exposure days.	[18]
CBNPs; GMD = 53 ± 1.57 nm	Male C57BL/6 mice; 12.5 μg/m^3^, nasal inhalation; 4 h/day, 7 days	The histopathology analyses revealed that the inhalation of nanoparticles exacerbated lung inflammation expressing a significant level of interleukin-6, interferon-γ, and fibronectin in lung tissues.	[93]
PAH-CBNPs; PS = 14.2 ± 0.1 nm, SSA = 115 ± 3 m^2^/g	Male Wistar rats (strain Crl: WI (Han)); 6 mg/m^3^, nasal inhalation; 6 h/day, 2 weeks	A significant increase in polymorphonuclear granulocyte numbers was observed for the animals treated with CBNPs and PAH-CBNPs when compared to clean air control on day 1 post-exposure. PAH-CBNPs induced bronchioalveolar hyperplasia, whereas CBNPs caused very slight histological alterations on day 14 post-exposure. When compared to control, only PAH-CBNPs exhibited significant IL-6 mRNA expression and keratinocyte chemoattractant.	[94]
C60; PS = 33 nm, SSA = 104.6 m^2^/g	Male Wistar rats; 0.33, 0.66 and 3.3 mg/kg, intratracheal instillation; 3 days, 1 week, 1, 3 and 6 months	No significant increase was observed in total cell count and in the expression of the cytokine-induced neutrophil chemoattractants CINC-1, -2αβ and -3 at a low dose of fullerene treated groups. The higher dose of fullerene treated rat group showed a significant increase in gene expression and total cell counts.	[95]
	Male Wistar rats; 0.12 ± 0.03 mg/m^3^, whole-body inhalation; 4 weeks, 6 h/day, 5 days/week	There were no significant changes in total cell count in BALF and gene expression of CINC-1, -2αβ and -3 in lung tissue.	
C60; GMD = 96 nm, SSA = 0.92 m^2^/g	Male Wistar rats; 0.12 mg/m^3^, whole-body inhalation; 4 weeks 6h/day, 5 days/week	Gene expression profiles revealed that the major histocompatibility complex (MHC) mediated immunity and metalloendopeptidase activity were upregulated at 3 days and 1 month of post-exposure. Some upregulated genes were involved in oxidative stress, inflammation, and apoptosis. The nanoparticles were found in alveolar epithelial cells and engulfed by macrophages.	[96]
C60; HD = 234.1 ± 48.9 nm and 856.5 ± 119.2 nm	gpt delta transgenic mice; 0.2 mg/animal, single dose, intratracheal instillation; 3 hMultiple doses (4 times)	Mutant frequencies were significantly increased (2–3 fold) in the lungs of the nanoparticle treated group when compared to control.There was a slight number of A:T to T:A transversion in C60 treated animals, while no genetic transversion was observed in control groups.	[67]
C60; PS = 46.7 ± 18.6 nm	ICR male mice; 0.5, 1, 2 mg/kg, intratracheal instillation; 1, 7, 14 and 28 days	Increase in pro-inflammatory cytokines including TNF-α, IL-1 and IL-6 and increase in T-cell distribution were observed in C60 treated mice. The gene expression of MHC class 2 was greater than that of MHC class 1 (H2-T23).	[97]
C60; HD = 407–5117 nm	Female Fisher 344 rats; single oral intragastric administration; 0.064 and 64 mg/kg; 24 h	Only high dose of fullerene generated oxidative damage by expressing a high level of mRNA 8-oxoguanine DNA glycosylase (8-oxodG) in the lung.	[98]
C60; n/a	Sprague-Dawley male and female rats; 2000 mg/kg, oral exposure, single dose; 14 days	No acute oral toxicity and no deaths were reported.	[26]
C60(OH)n	BALB/c female mice; 0.02, 0.2, 2.0, 20 and 200 μg/animal, intratracheal instillation; 24 h	The BAL data indicated that only 200 μg treated mice showed increased neutrophil influx in the lungs causing inflammation, whereas other low concentration treated groups did not present any significant changes.	[99]
SWCNTs; L ≤ 1 µm, W = 0.9–1.7 nm	Female Fisher 344 rats; 0.064 and 64 mg/Kg, single dose, oral intragastric administration; 24 h	SWCNTs were reported to cause oxidatively damaged DNA in lung and liver by increasing the level of 8-oxodG.	[98]
SWCNTs; n/a	Male Sprague-Dawley rats; 0.4, 2 and 4 mg/kg, intrapulmonary instillation; 1, 7, 30 and 90 days	Increase in lung granulomatous and inflammatory responses along with fibrosis and collagen deposition was observed in a time and dose-dependent manner for SWCNTs treated groups.	[41]
SWCNTs; L = 10 nm to several µm, W = 1–2 nm	Male ICR mice; 0.5 mg/kg, intratracheal instillation, single dose; 3 and 14 days	The histological data of SWCNTs treated groups revealed that an increase in macrophage infiltration, foamy-like macrophages formation in the alveolar space, and no significant granuloma formation were observed at 3 days of investigation. Meanwhile, a profound multifocal granuloma was found after 14 days.	[40]
SWCNTs; n/a	Female C57BL/6 mice; 40 µg/mouse, single dose, intraperitoneal injection; 1 and 7 days	Non-degraded nanotubes treated mice induced inflammation and tissue granulomas, while biodegraded nanotubes treated mice were not induced.	[100]
SWCNTs; L ≤ 5 µm, W = ~8 nm	SPF male and female Wistar rats; 2 and 10 mg/kg, intratracheal instillation; 5 weeks	High dose exposure of SWCNTs registered increased level of inflammatory markers such as IL-1, IL-6 and TNF-α in BALF than low dose exposure in rat lungs. Transgelin 2 gene expression was also found to be higher in high dose treated rats.	[101]
SWCNTs; HD = 48.4 nm	Male ICR mice; 25, 50 and 100 μg/kg, intratracheal instillation; after 24 h	The administration of SWCNTs increased the secretion of IL-6 and MCP-1, and the number of total cells including neutrophils, lymphocytes, and eosinophils in the lungs of higher dose-treated mice.	[39]
SWCNTs; L = ≤1 µm, W = 0.8–1.7 nm	Female C57BL/6J mice; 0.9, 2.8, 8.4 mg/kg, intratracheal instillation, single dose; 1, 3 and 28 days	A dose-dependent increase in Saa3 mRNA expression was observed in the lung.	[102]
SWCNTs; PS = 1–2 nm, SSA = 1040 m^2^/g	Female C57BL/6J mice; 40 μg/mouse, pharyngeal aspiration, single dose; 1, 7 and 28 days	The SWCNTs treated vitamin E-deficient mice had shown a greater decrease in pulmonary antioxidants when compared to controls. Acute inflammation and enhanced profibrotic responses were also observed.	[42]
SWCNTs; L = ≤1 µm, W = 0.8–1.2 nm, SSA = 400–1000 m^2^/g	Male C57BL/6J mice; 10 μg/mouse, pharyngeal aspiration, single dose; 2 weeks	Both Survanta (natural lung surfactant) dispersed and acetone/sonication dispersed SWCNTs induced lung fibrosis in mice by increasing collagen deposition.	[43]
MWCNTs; PS = 15–50 nm	Male Wistar rats; 5mg/m^3^, nasal inhalation; single dose; 4 h, 1, 7, and 14 days	A significant increase in cell count, lactate dehydrogenase, alkaline phosphatase, and cytokines and a decrease in cell viability and alveolar macrophage count were observed in MWCNTs-treated rats in all the investigated days, when compared to control rats. Inflammation, granuloma, and fibrosis were also reported in the lungs of MWCNTs-treated rats on 7 and 14 days of post-exposure.	[32]
MWCNTs; short (L = 1–5 µm, W = 15 ± 5 nm), intermediate (L = 5–20 µm, W = 15 ± 5 nm), long (L = ~13 µm, W = 40–50 nm)	Female C57Bl/6 mice; 50 mg/mouse, intraperitoneal injection; 1 and 7 days	Size-dependent studies revealed that long sized MWCNTs (mean 13 µm) affected significant inflammation and granuloma in mice at 1 and 7 days of post-operation while short (1–5 µm) and intermediate (5–20 µm) MWCNTs did not cause any significant changes. Furthermore, short MWCNTs were readily involved in phagocytosis while long sized MWCNTs had frustrated phagocytosis.	[103]
MWCNTs; L = 1.1 ± 2.7 µm, W = 63 ± 1.5 nm	Male Wistar rats; 0.66 and 3.3 mg/kg, intratracheal instillation; 3, 7, 30, 90, and 180 days Male Wistar rats; whole-body inhalation; 6 h/day, 4 weeks	Lung inflammations and CINC-1 expressions were found significantly in high dose treated rats and temporary inflammation was observed in the low dose treated groups. Minimal pulmonary inflammation and a temporary increase in CINC-1 to CINC-3 expressions were found.	[104]
MWCNTs; L = 5.9 ± 0.05 µm, W = 9.7 ± 2.1 nm, SSA = 378 ± 20 m^2^/g	Female Sprague–Dawley rats; 0.5 and 2 mg/rat, intratracheal instillation; 0, 28 and 60 days	At 60 days, pulmonary lesions were observed for MWCNTs treated rats owing to collagen-rich granulomas formation protruding in the bronchial lumen. TNF-α was excessively produced in the lungs of treated animals.	[105]
MWCNTs; n/a	Male guinea pigs; 12.5 mg/pig, intratracheal instillation; 90 days	At 90 days, the MWCNTs exposure caused pneumonitis with mild peribronchiolar fibrosis in pigs, which was not observed in the controls.	[106]

Abbreviations: PS, particle size; IS, interlayer spacing; HD, hydrodynamic diameter; GMD, geometric mean diameter; SSA, specific surface area; L, length; W, width; n/a, not available.
